# Early Leukocyte Profiles in Preterm Infants Born to Mothers with Systemic Lupus Erythematosus: An Exploratory Matched Cohort Study

**DOI:** 10.3390/children13091237

**Published:** 2026-09-12

**Authors:** Hikaru Takahashi, Shutaro Suga, Toshihiko Manabe, Yusuke Saito, Reiji Fukano

**Affiliations:** Department of Pediatrics, School of Medicine, University of Occupational and Environmental Health, Kitakyushu 807-8555, Japan; t-hikaru@med.uoeh-u.ac.jp (H.T.); mnbtoshi7663@med.uoeh-u.ac.jp (T.M.); ysaitoh0256@med.uoeh-u.ac.jp (Y.S.); fukano@med.uoeh-u.ac.jp (R.F.)

**Keywords:** systemic lupus erythematosus, preterm infant, maternal SLE flare, neutropenia, leukopenia, neonatal hematology

## Abstract

**Highlights:**

**What are the main findings?**

• Matched-set analyses showed no clear overall differences in hematologic profiles between SLE-exposed preterm infants and maturity-matched controls.
• In exploratory flare-stratified analyses, WBC and neutrophil nadirs were lower in the maternal-flare subgroup than in its corresponding matched controls.

**What are the implications of the main findings? **

• Maternal SLE flare may be associated with an early laboratory signal of lower neonatal leukocyte nadirs.
• The clinical significance of this exploratory finding remains uncertain and requires confirmation in larger multicenter cohorts.

**Abstract:**

**Background/Objectives**: Maternal systemic lupus erythematosus (SLE) is associated with preterm birth, but whether maternal disease activity influences early neonatal hematologic profiles beyond developmental immaturity remains unclear. We compared hematologic parameters between SLE-exposed preterm infants and maturity-matched controls and explored findings according to maternal SLE flare during pregnancy. **Methods**: This single-center retrospective matched-cohort study included 13 SLE-exposed preterm infants and 39 controls matched 1:3 by gestational age, birth weight, and sex. White blood cell (WBC) count, absolute neutrophil count (ANC), hemoglobin concentration, and platelet count were assessed at birth, around postnatal day 5, and at their nadir during hospitalization. Group differences were estimated using linear regression models including matched-set identifiers as fixed effects. Flare-stratified and hepatic analyses were exploratory, without adjustment for multiple comparisons. **Results**: Overall matched-set analyses showed no clear differences in WBC count, ANC, hemoglobin, or platelet values between SLE-exposed infants and controls. The estimated difference in nadir ANC was −405/μL (95% confidence interval [CI], −985 to 174/μL; *p* = 0.164). In the maternal-flare subgroup, nadir WBC count was lower than in its matched controls (difference, −2.31 × 10^3^/μL; 95% CI, −3.96 to −0.65 × 10^3^/μL; *p* = 0.009), as was nadir ANC (difference, −672/μL; 95% CI, −1260 to −85/μL; *p* = 0.028). Corresponding matched-set differences were not evident at birth or around postnatal day 5. The non-flare subgroup did not show a consistent pattern of leukocyte suppression. **Conclusions**: Maternal SLE exposure was not clearly associated with an overall difference in neonatal hematologic profiles within matched sets. Maternal SLE flare may identify a subgroup with lower WBC and neutrophil nadirs, but this exploratory finding requires confirmation in larger studies.

## 1. Introduction

Systemic lupus erythematosus (SLE) is a multisystem autoimmune disease that predominantly affects women of reproductive age. Pregnancies complicated by SLE are associated with increased risks of preterm birth and other adverse perinatal outcomes, particularly when maternal disease is active during gestation [1,2,3,4]. Current clinical recommendations therefore emphasize the assessment of maternal disease activity and serologic status during pregnancy. However, the relationship between maternal SLE activity and early neonatal hematologic findings remains poorly defined.

Interpretation of neonatal hematologic parameters is particularly challenging in preterm infants. Complete blood cell counts vary substantially according to gestational and postnatal age because of developmental hematopoiesis and early postnatal adaptation [5,6]. Hematologic abnormalities in infants born to mothers with SLE have primarily been described in the context of neonatal lupus and transplacental autoantibody exposure, often in term or heterogeneous neonatal populations [7]. A recent cohort study characterized thrombocytopenia in preterm infants born to mothers with SLE, but did not include preterm infants without maternal autoimmune disease as maturity-matched controls or specifically examine early leukocyte profiles according to maternal disease activity [8]. The primary objective of this study was to determine whether maternal SLE exposure was associated with differences in hematologic profiles among preterm infants compared with maturity-matched controls while retaining the matched-set structure in the analysis. Separately, we conducted hypothesis-generating analyses to describe hematologic profiles according to maternal SLE flare during pregnancy. These flare-stratified analyses were exploratory and were not designed to establish effect modification by maternal disease activity.

## 2. Materials and Methods

### 2.1. Study Design and Ethics

This single-center retrospective matched-cohort study was conducted at the Neonatal Intensive Care Unit (NICU) of the University of Occupational and Environmental Health (UOEH) Hospital, Japan. Eligible infants admitted between 1 January 2010 and 31 December 2024 were reviewed. The study protocol was approved by the Institutional Review Board of UOEH (Approval No. CR25-129).

### 2.2. Study Population

Electronic medical records were screened to identify neonates born to mothers with a confirmed diagnosis of systemic lupus erythematosus (SLE). Inclusion criteria were preterm birth (gestational age [GA] < 37 weeks) and NICU admission. Exclusion criteria were term birth (GA ≥ 37 weeks), absence of NICU admission, and neonatal death before completion of early hematologic evaluation.

**Figure 1:**
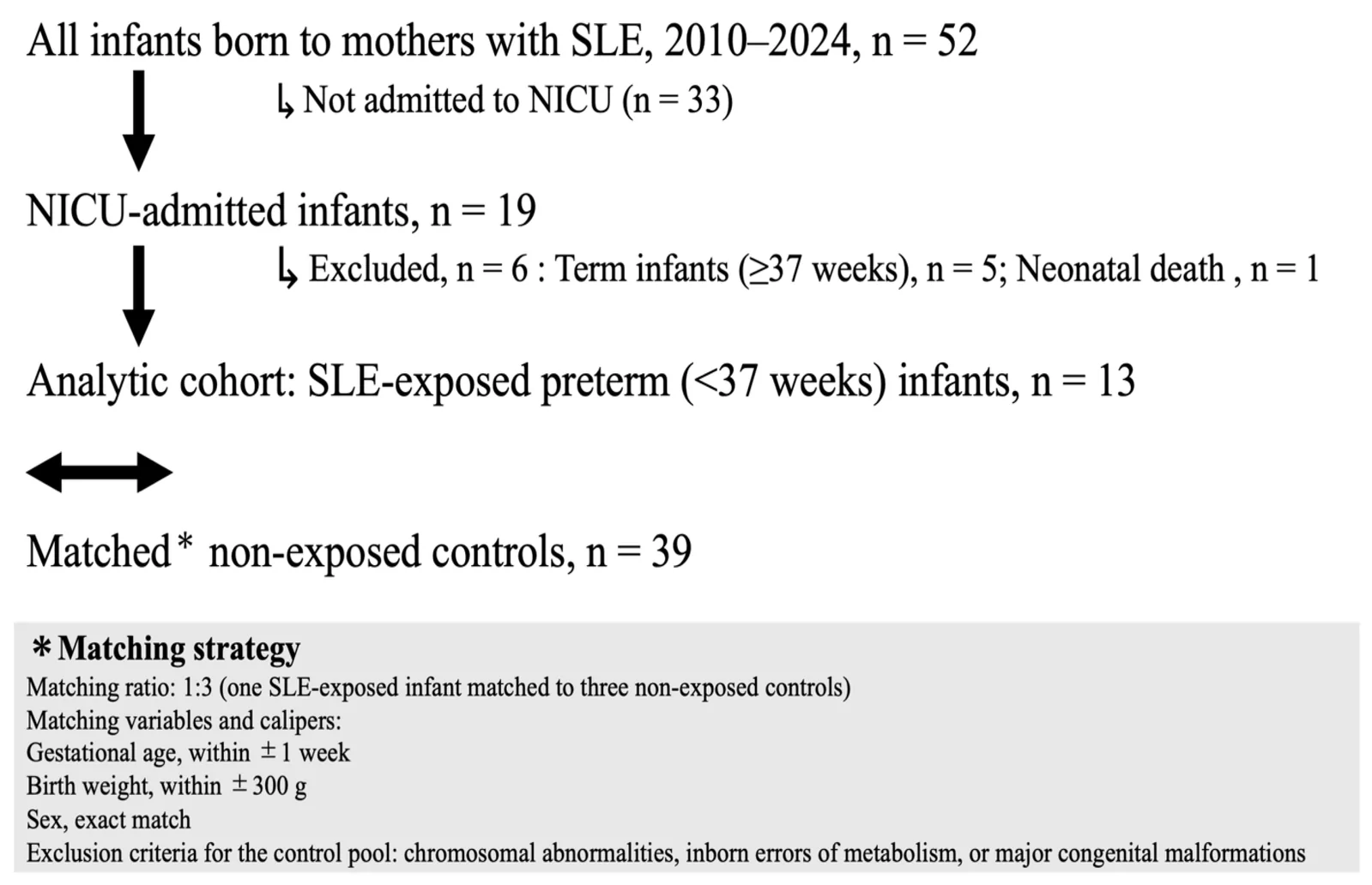
Study cohort selection and matching strategy for maternal SLE-exposed and non-exposed preterm infants. SLE, systemic lupus erythematosus; NICU, neonatal intensive care unit.

Among 52 neonates born to mothers with SLE who were initially identified, 13 preterm infants met the eligibility criteria and were included in the SLE-exposed group (Figure 1).

### 2.3. Matched Control Selection

For each SLE-exposed infant, three control infants were selected from preterm infants admitted to the same NICU during the same study period. Control infants were born to mothers without a documented autoimmune disease and otherwise fulfilled the same eligibility criteria as the SLE-exposed infants.

Controls were matched according to GA within ±1 week, birth weight (BW) within ±300 g, and identical sex. When more than three eligible infants met these criteria, those with the closest GA and BW were selected. This procedure yielded 39 matched control infants. Controls were selected without replacement, and matching was performed without reference to hematologic outcomes.

Matching was used to improve clinical comparability between groups. Because infants within each matched set were selected according to the same maturity criteria, the primary analyses retained matched-set membership explicitly rather than treating all observations as independent.

### 2.4. Data Collection

Neonatal demographic and clinical variables were extracted from electronic medical records. Maternal clinical data included SLE disease activity, serologic findings, pregnancy complications, and medication exposure. Maternal anti-Ro autoantibody status was abstracted from the clinical laboratory records. Current consensus recommendations distinguish anti-TROVE2/Ro60 from anti-TRIM21/Ro52 autoantibodies [9]. However, the historical laboratory records used in this study reported these results as anti-SSA/Ro without consistently distinguishing the antigen specificity. We therefore use the broader term anti-Ro autoantibodies throughout the manuscript. Neonatal clinical outcomes and laboratory data were collected longitudinally throughout hospitalization. Maternal characteristics were summarized at the infant level to align with the neonatal cohort; consequently, shared maternal characteristics were repeated for included siblings from the same multiple pregnancy (Supplementary Table S3).

### 2.5. Outcomes

The primary outcomes were white blood cell (WBC) count, absolute neutrophil count (ANC), hemoglobin concentration, and platelet count. Values at birth were defined as the first measurements obtained within 24 h after delivery; postnatal day 5 values were measurements obtained on day 5 (±1 day); and nadirs were the lowest recorded values during the entire NICU hospitalization. The postnatal day on which each nadir occurred was also recorded. Secondary outcomes included neonatal morbidities and clinical course during hospitalization. Exploratory hepatic outcomes included total bilirubin, aspartate aminotransferase (AST), and alanine aminotransferase (ALT) at birth, around postnatal day 5, and at their peak during hospitalization.

### 2.6. Maternal SLE Flare and Stratified Analysis

Maternal clinical records, disease activity scores, and treatment changes during pregnancy were reviewed to classify the corresponding pregnancies according to maternal SLE flare status. Maternal SLE flare was defined as an increase of at least 4 points in the Systemic Lupus Erythematosus Disease Activity Index (SLEDAI) score from the prepregnancy baseline and/or clinician-documented worsening of SLE requiring treatment escalation [10,11]. The six flare-exposed infants arose from five pregnancies, including one multiple pregnancy. All five pregnancies met the SLEDAI-based criterion; two also had clinician-documented worsening requiring treatment escalation and therefore met both components of the flare definition. Treatment escalation consisted of rituximab administration and intravenous methylprednisolone pulse therapy combined with cyclosporine, each in one pregnancy.

The SLE-exposed group was divided into flare (n = 6) and non-flare (n = 7) subgroups. For the stratified analyses, the matched sets corresponding to each subgroup were retained, yielding 18 controls for the flare subgroup and 21 controls for the non-flare subgroup. Each subgroup was evaluated relative to its own maturity-matched controls. Direct comparisons between the flare and non-flare infants were considered descriptive because the two SLE-exposed subgroups were not maturity-matched to each other.

### 2.7. Statistical Analysis

Continuous variables are presented as medians and interquartile ranges, and categorical variables as numbers and percentages. Neonatal characteristics in Table 1 were summarized descriptively without formal hypothesis testing. The comparison between the overall SLE-exposed group and its matched controls was considered the main analysis. Analyses of individual hematologic parameters and time points, hepatic outcomes, maternal-flare subgroups, and maternal anti-Ro autoantibody status were considered exploratory.

For hematologic and hepatic outcomes, matched-set fixed-effects linear regression models were fitted with the outcome as the dependent variable, maternal SLE exposure as the group indicator, and matched-set identifiers as fixed effects. The coefficient for SLE exposure represents the estimated within-set mean difference between the SLE-exposed group and controls and is reported with its 95% confidence interval (CI). Analyses were based on available cases, and no missing values were imputed; therefore, the analytic sample size could vary by outcome.

For analyses stratified according to maternal SLE flare during pregnancy, the same fixed-effects approach was applied separately to the flare and non-flare matched sets. Direct comparisons between flare and non-flare infants were performed using a two-sided exact permutation Mann–Whitney U test. Because these two subgroups differed substantially in gestational age and birth weight and were not directly matched to each other, these comparisons were used only to describe subgroup characteristics and were not interpreted as estimates of the effect of maternal flare. The subgroup-specific matched-set estimates were not formally compared; therefore, effect modification by maternal flare was not established.

All statistical tests were two-sided. No formal adjustment was made for multiple comparisons. A *p* value < 0.05 was considered nominally statistically significant; however, unadjusted *p* values and 95% CIs are reported, and all outcome-specific and subgroup findings should be interpreted as exploratory and hypothesis-generating. Analyses were performed using JMP version 19.2.0 (JMP Statistical Discovery LLC, Cary, NC, USA).

## 3. Results

### 3.1. Study Population and Clinical Characteristics

The final matched cohort comprised 52 preterm infants: 13 infants born to mothers with systemic lupus erythematosus (SLE-exposed group) and 39 matched control infants. The median gestational age (GA) was 30 weeks (interquartile range [IQR], 28–32 weeks) in both groups. Median birth weight was 1286 g (IQR, 958–1453 g) in the SLE-exposed group and 1244 g (IQR, 964–1445 g) in the control group (Table 1).

Other neonatal characteristics, including small-for-gestational-age status, 5-min Apgar score, time to establishment of enteral feeding, weight gain, discharge weight, major neonatal morbidities, and clinical course, are presented descriptively in Table 1.

Among the 13 SLE-exposed infants, 6 (46.2%) were born following a pregnancy complicated by maternal SLE flare. Corresponding maternal lupus nephritis, anti-Ro autoantibody positivity, and hydroxychloroquine exposure were recorded for 12 (92.3%), 11 (84.6%), and 10 (76.9%) infants, respectively (Supplementary Table S3).

### 3.2. Matched-Set Hematologic Analysis in the Overall Cohort

Matched-set fixed-effects analyses did not show clear overall differences between SLE-exposed infants and controls in WBC count, ANC, platelet count, or hemoglobin at birth, around postnatal day 5, or at their nadir during hospitalization (Table 2). For nadir ANC, the medians were 924/μL (IQR, 594–1590/μL) and 1716/μL (IQR, 1098–2204/μL), respectively; the within-set mean difference was −405/μL (95% CI, −985 to 174/μL; *p* = 0.164).

**Table 2:** Matched-Set Analysis of Neonatal Hematologic Profiles According to Maternal SLE Exposure.

Parameter	SLE-Exposed Infants Median (IQR)	Maturity-Matched Controls Median (IQR)	Matched-Set Difference (95% CI) †	*p* Value
Parameters at Birth (Day 0)				
White blood cell count (×10^3^/μL)	6.4 (2.4–10.6)	8.25 (6–11.43)	−1.92 (−5.96–2.13)	0.343
Absolute neutrophil count (/μL)	2139 (702–5920)	2783 (1335–4163)	−548 (−3423–2327)	0.700
Platelet count (×10^3^/μL)	231 (214–285)	249.5 (172.2–296.8)	43.3 (−4.9–91.4)	0.077
Hemoglobin (g/dL)	16.4 (14.4–17.3)	16.7 (15.03–18.38)	−1.34 (−2.92–0.23)	0.093
Parameters at Postnatal Day 5				
White blood cell count (×10^3^/μL)	6.8 (6.3–9.7)	8.25 (5.35–10.35)	−0.73 (−2.96–1.50)	0.507
Absolute neutrophil count (/μL)	3264 (1780–4405)	3214 (1978–5239)	−548 (−2151–1056)	0.488
Platelet count (×10^3^/μL)	248 (173–316)	242 (159.2–327.8)	13.8 (−57.3–84.8)	0.694
Hemoglobin (g/dL)	13.4 (10.9–17.3)	13.05 (11.53–14.57)	0.50 (−0.96–1.97)	0.486
Nadir During Hospitalization				
White blood cell count (×10^3^/μL)	5.4 (2.4–8.4)	6 (4.85–8.03)	−0.66 (−1.79–0.47)	0.242
Postnatal day at nadir	5 (0–69)	2.5 (0–21.2)	10.3 (−8.1–28.8)	0.263
Absolute neutrophil count (/μL)	924 (594–1590)	1716 (1098–2204)	−405 (−985–174)	0.164
Postnatal day at nadir	5 (0–11)	0 (0–5)	2.3 (−14.0–18.7)	0.774
Platelet count (×10^3^/μL)	229 (140–285)	214 (135–277.5)	5.5 (−41.5–52.5)	0.815
Postnatal day at nadir	5 (0–11)	0 (0–4)	7.0 (0.9–13.0)	0.025 *
Hemoglobin (g/dL)	9.7 (8.5–11.1)	9.5 (8.8–11.35)	−0.56 (−1.53–0.40)	0.245
Postnatal day at nadir	29 (20–48)	30.5 (18.2–45.2)	3.1 (−11.9–18.0)	0.676

Data are presented as median (interquartile range [IQR]). † Matched-set differences represent the coefficient for maternal SLE exposure (SLE-exposed minus control) from linear regression models including matched-set identifiers as fixed effects and are expressed in the units of each outcome. Analyses used available cases; corresponding sample sizes are shown as SLE/control. All tests were two-sided. No adjustment was made for multiple comparisons; results should therefore be interpreted as exploratory. * *p* < 0.05. Values at birth were obtained within 24 h after delivery; day 5 values were measured on postnatal day 5 (±1 day); and nadirs were the lowest values recorded during NICU admission. SLE, systemic lupus erythematosus; CI, confidence interval; NICU, neonatal intensive care unit.

The timing of the WBC and ANC nadirs also did not differ clearly between groups in the matched-set models. The only nominally significant overall timing result was a later platelet nadir in SLE-exposed infants (within-set mean difference, 7.0 days; 95% CI, 0.9–13.0 days; *p* = 0.025). Because this was one of multiple exploratory comparisons, it was not interpreted as a primary finding.

In exploratory hepatic analyses, total bilirubin at birth was higher in SLE-exposed infants than in controls (within-set mean difference, 0.47 mg/dL; 95% CI, 0.14–0.80 mg/dL; *p* = 0.006), whereas total bilirubin at later time points and AST and ALT values did not show a consistent pattern of differences (Supplementary Table S1). Stratified hepatic analyses yielded nominally higher total bilirubin at birth and around postnatal day 5 in the non-flare subgroup than in its matched controls, but these findings were among multiple exploratory comparisons without adjustment for multiplicity (Supplementary Table S2).

### 3.3. Exploratory Analyses According to Maternal SLE Flare

Six infants in the maternal-flare subgroup were evaluated with 18 matched controls, and 7 infants in the non-flare subgroup were evaluated with 21 matched controls. The flare infants were substantially more premature and had lower birth weight than the non-flare infants (median gestational age, 27.5 vs. 32 weeks, *p* = 0.001; median birth weight, 852 vs. 1453 g, *p* = 0.002); therefore, direct comparisons between these subgroups were not interpreted as flare effects. Within the flare matched sets, nadir WBC count was lower than in controls (2.35 × 10^3^/μL [IQR, 2.23–2.48 × 10^3^/μL] vs. 4.8 × 10^3^/μL [IQR, 3.47–6.2 × 10^3^/μL]; within-set mean difference, −2.31 × 10^3^/μL; 95% CI, −3.96 to −0.65 × 10^3^/μL; *p* = 0.009). Nadir ANC was also lower (634/μL [IQR, 527–702/μL] vs. 1302/μL [IQR, 1012–1716/μL]; difference, −672/μL; 95% CI, −1260 to −85/μL; *p* = 0.028). WBC and ANC nadirs occurred on the day of birth in all six flare infants, but the matched-set differences in nadir timing were not statistically significant. By postnatal day 5, median WBC count and ANC in the flare subgroup were numerically higher than the corresponding values at birth (WBC count, 4.9 vs. 2.35 × 10^3^/μL; ANC, 1450 vs. 696/μL). Although the day 5 within-set estimates remained lower than those of the matched controls, the 95% confidence intervals included zero (WBC count: mean difference, −1.89 × 10^3^/μL; 95% CI, −5.42 to 1.65 × 10^3^/μL; *p* = 0.269; ANC: mean difference, −1517/μL; 95% CI, −4558 to 1524/μL; *p* = 0.296). At birth, the confidence intervals for the corresponding matched-set estimates also included zero. The non-flare subgroup showed no consistent pattern of lower leukocyte values relative to its controls; nominal findings involving higher admission ANC and platelet count and later nadir timing were isolated among multiple exploratory comparisons (Table 3).

**Table 3:** Exploratory Neonatal Hematologic Profiles According to Maternal SLE Flare Status and Maturity-Matched Controls.

Parameter	(A) Flare (n = 6)	(B) Non-Flare (n = 7)	P_1_	(C) Controls for A	A vs. C: Within-Set Mean Difference (95% CI) †	P_2_	(D) Controls for B	B vs. D: Within-Set Mean Difference (95% CI) †	P_3_
Neonatal Characteristics									
Gestational age, weeks	27.5 (27–28)	32 (32–33)	0.001 *	27 (26.2–28)	—	—	32 (32–33)	—	—
Birth weight, g	852 (712–974)	1453 (1335–1566)	0.002 *	957 (696–1094)	—	—	1401 (1335–1698)	—	—
Parameters at Birth (Day 0)									
White blood cell count (×10^3^/μL)	2.35 (2.23–2.48)	10.6 (10.15–14.05)	0.001 *	6.6 (4.88–10.95)	−6.61 (−14.51–1.28)	0.095	9.6 (7.7–11.27)	2.17 (−0.58–4.91)	0.115
Absolute neutrophil count (/μL)	696 (512–704)	5880 (4206–6371)	0.003 *	2118 (1051–6151)	−4187 (−10,257–1883)	0.160	3232 (1872–4105)	2120 (158–4082)	0.036 *
Platelet count (×10^3^/μL)	206.5 (167.5–219.2)	285 (247.5–399)	0.005 *	202.5 (137.2–254.8)	−9.8 (−58.4–38.7)	0.674	272.5 (206.8–301.8)	89.5 (11.2–167.8)	0.027 *
Hemoglobin (g/dL)	14.75 (14.45–16.02)	17 (14.65–17.5)	0.628	16.3 (15–18.8)	−1.45 (−4.00–1.10)	0.247	16.7 (15.1–17.75)	−1.25 (−3.40–0.91)	0.242
Parameters at Postnatal (Day 5)									
White blood cell count (×10^3^/μL)	4.9 (3.05–6.6)	9.7 (9.15–11.9)	0.002 *	5.3 (4.5–7.95)	−1.89 (−5.42–1.65)	0.269	9.85 (8.22–11.55)	0.32 (−2.78–3.42)	0.826
Absolute neutrophil count (/μL)	1450 (1225–1890)	4224 (3264–5824)	0.018 *	2272 (1612–3921)	−1517 (−4558–1524)	0.296	3912 (3020–5239)	193 (−1646–2032)	0.824
Platelet count (×10^3^/μL)	177 (139.2–228.2)	316 (277.5–452.5)	0.014 *	211.5 (151.8–282.2)	−44.5 (−136.7–47.7)	0.316	289.5 (181.8–354.2)	66.7 (−44.4–177.7)	0.217
Hemoglobin (g/dL)	12.9 (11.38–13.3)	15.2 (13.05–17.45)	0.252	11.8 (9.85–13.4)	0.88 (−1.37–3.13)	0.414	13.9 (12.93–16.1)	0.16 (−2.02–2.35)	0.874
Nadir During Hospitalization									
White blood cell count (×10^3^/μL)	2.35 (2.23–2.48)	8.4 (7.6–9.65)	0.001 *	4.8 (3.47–6.2)	−2.31 (−3.96–−0.65)	0.009 *	7.6 (5.95–9.83)	0.77 (−0.59–2.13)	0.252
Postnatal day at nadir	0 (0–0)	5 (5–71)	0.006 *	1 (0–5)	−8.9 (−42.3–24.6)	0.575	4 (0–23.5)	25.7 (4.8–46.5)	0.019 *
Absolute neutrophil count (/μL)	634 (527–702)	1590 (1108–2994)	0.014 *	1302 (1012–1716)	−672 (−1260–−85)	0.028 *	1944 (1510–2944)	−173 (−1181–835)	0.722
Postnatal day at nadir	0 (0–0)	5 (5–41)	0.033 *	0 (0–5)	−18.2 (−44.9–8.5)	0.163	0 (0–5)	19.0 (−0.1–38.1)	0.051
Platelet count (×10^3^/μL)	132 (101.5–161.8)	285 (246.5–339)	0.008 *	196 (124.8–213.8)	−41.6 (−103.4–20.3)	0.174	271.5 (201.8–301.8)	46.4 (−23.0–115.9)	0.178
Postnatal day at nadir	5.5 (5–9.8)	0 (0–17)	0.224	1 (0–5)	6.6 (−5.0–18.1)	0.241	0 (0–1)	7.3 (0.2–14.4)	0.043 *
Hemoglobin (g/dL)	8.8 (8.35–10.07)	9.8 (9.35–11.15)	0.312	9.1 (8.5–9.7)	−0.16 (−1.61–1.30)	0.825	10.1 (9.38–12.18)	−0.92 (−2.31–0.47)	0.183
Postnatal day at nadir	21 (6.2–56.8)	29 (27–38.5)	0.703	23 (12–71)	0.8 (−34.4–35.9)	0.963	31 (29–43)	4.9 (−6.0–15.9)	0.354

Data are presented as median (interquartile range [IQR]). P_1_ compares the flare and non-flare groups among SLE-exposed infants using a two-sided exact permutation Mann–Whitney U test. † Within-set mean differences represent the coefficient for SLE exposure (SLE subgroup minus its corresponding control) from linear regression models including matched-set identifiers as fixed effects; P_2_ and P_3_ are the corresponding model *p* values. Available cases were analyzed, with n shown as subgroup/control. No model estimates were calculated for gestational age or birth weight because these were matching variables. No adjustment was made for multiple comparisons; all analyses were exploratory. * *p* < 0.05. SLE, systemic lupus erythematosus; CI, confidence interval.

Maternal anti-Ro autoantibodies were documented for 11 of the 13 SLE-exposed infants, corresponding to 10 of 12 pregnancies. Neonatal hematologic and hepatic profiles according to maternal anti-Ro autoantibody status are presented descriptively in Supplementary Table S4.

## 4. Discussion

In this matched cohort, fixed-effects analyses retaining the matched-set structure did not provide clear evidence that maternal SLE exposure was associated with an overall difference in neonatal WBC count, ANC, platelet count, or hemoglobin. In exploratory flare-stratified analyses, infants born following maternal SLE flare had lower WBC and ANC nadirs than their matched controls. However, corresponding matched-set differences were not evident at birth or around postnatal day 5. Given the small subgroup size, these findings should be regarded as hypothesis-generating.

Hematologic findings in preterm infants must be interpreted in relation to developmental maturity. WBC and neutrophil reference intervals vary according to gestational and postnatal age, and blood cell counts change rapidly during the first days after birth [5,6,12]. The flare and non-flare infants in this cohort differed markedly in gestational age and birth weight, underscoring why unadjusted comparisons between these two SLE-exposed subgroups cannot isolate an effect of maternal disease activity.

Previous studies of hematologic abnormalities in infants born to mothers with SLE have focused primarily on neonatal lupus and transplacental autoantibody exposure [7]. A recent cohort study of 154 preterm infants born to mothers with SLE reported thrombocytopenia in approximately one-third of infants and identified gestational age, maternal hypertensive disorders of pregnancy, and hydroxychloroquine use as associated factors [8]. That study did not include preterm infants without maternal autoimmune disease as maturity-matched controls. In the present cohort, matched-set estimates did not indicate an overall difference in platelet values, although platelet nadir occurred nominally later in the SLE-exposed group. This isolated timing result requires cautious interpretation because multiple outcomes and time points were examined.

The flare-stratified results were most consistent for nadir leukocyte measures: WBC and ANC nadirs were lower in the flare subgroup than in its matched controls, whereas estimates at birth and around postnatal day 5 did not exclude no difference. The non-flare subgroup did not show a corresponding pattern of leukocyte suppression. Nevertheless, statistical significance in one subgroup and not in another does not establish a difference between subgroup effects. No formal interaction test was performed to assess effect modification, and the direct flare versus non-flare comparisons were strongly confounded by maturity. Other nominal findings—including higher admission ANC and platelet count in the non-flare subgroup, later nadir timing for selected outcomes, and differences in total bilirubin—did not form a consistent hematologic pattern and may reflect multiple testing.

The temporal pattern provides additional context. In all six infants in the maternal-flare subgroup, the WBC and ANC nadirs occurred on the day of birth. By postnatal day 5, median WBC count and ANC were numerically higher than the corresponding values at birth. Although the day 5 within-set estimates remained lower than those of the matched controls, their 95% confidence intervals included zero. This pattern is consistent with attenuation of the early leukocyte reduction rather than persistent hematologic suppression, although within-infant changes over time were not formally analyzed. Admission counts and subsequent nadirs may be influenced by delivery-related stress, the timing and frequency of blood sampling, antenatal corticosteroid exposure, maternal hypertension or placental dysfunction, and maternal or neonatal treatment. Regression to the mean and clinically determined laboratory testing may also have contributed to the observed pattern.

Several biological mechanisms could potentially underlie an association between maternal disease activity and neonatal leukocyte nadirs. Neonates have a smaller neutrophil storage pool than older children and adults, and neutrophil production and functional responses are less mature in preterm infants [13,14]. This limited myeloid reserve may increase susceptibility to antenatal inflammatory or placental influences. Transplacental anti-Ro autoantibodies may also contribute to neonatal hematologic abnormalities [7]. Maternal anti-Ro positivity was frequent in this cohort. However, the exploratory anti-Ro-stratified analysis was markedly imbalanced, with 11 anti-Ro-positive and only two anti-Ro-negative infants, precluding meaningful assessment of whether maternal anti-Ro exposure was associated with the observed hematologic or hepatic profiles. Moreover, antigen-specific results for anti-TROVE2/Ro60 and anti-TRIM21/Ro52 were not separately available. Maternal immune activation may influence fetal hematopoietic regulation [15,16], but neither the direction nor the clinical importance of such an effect can be determined from the present data. Maternal hematologic abnormalities and medication exposure are additional possible explanations.

From a clinical perspective, maternal SLE flare may be considered as one element of the clinical context when unexpectedly low WBC or neutrophil values are identified in a preterm infant; however, the present exploratory findings do not support flare-specific monitoring recommendations. Nevertheless, neutropenia is common among preterm infants and may also be associated with maternal conditions such as pregnancy-induced hypertension [13,17]. Low ANC values should therefore be interpreted alongside gestational age, postnatal age, maternal complications, sampling history, and the infant’s clinical condition. The lower leukocyte nadirs were followed by numerically higher WBC and ANC values by postnatal day 5, and no clinical treatment interventions were attributed specifically to leukopenia or neutropenia. Nevertheless, the study was not powered to exclude associations with uncommon adverse clinical outcomes. Thus, the observed differences appear to primarily represent laboratory findings of uncertain clinical significance.

This study has several strengths. It examined a rare neonatal population over an extended period, used maturity-matched preterm controls, retained matched-set membership in the statistical models, and incorporated maternal disease activity rather than treating maternal SLE as a uniform exposure. However, several limitations should be acknowledged. First, only 13 SLE-exposed infants were included, of whom six were born following maternal flare; estimates were therefore imprecise and generalizability was limited. Second, multiple hematologic, timing, subgroup, and hepatic comparisons were performed without formal adjustment for multiplicity, so type I error cannot be excluded. Third, the flare and non-flare subgroups differed substantially in maturity, were evaluated against separate matched controls, and were not compared using a formal interaction model; effect modification cannot be established. Fourth, retrospective classification of maternal flare may have been affected by variation in the timing and completeness of disease activity assessments. Finally, this was a single-center study restricted to preterm infants admitted to the NICU, and the findings may not generalize to term infants or infants not requiring intensive care.

## 5. Conclusions

Matched-set analyses did not provide clear evidence of an overall association between maternal SLE exposure and neonatal hematologic profiles. In exploratory flare-stratified analyses, lower WBC and ANC nadirs were observed among infants born following maternal SLE flare compared with their matched controls, whereas differences were not evident at birth or around postnatal day 5. These findings suggest a possible association between maternal disease activity and neonatal leukocyte nadirs but do not establish causality. Larger multicenter studies incorporating standardized maternal disease assessment, neonatal blood-sampling schedules, and relevant maternal and placental covariates are needed to confirm this observation.

## Figures and Tables

**Table 1:** Baseline Characteristics and Neonatal Outcomes According to Maternal SLE Exposure.

Characteristic	SLE-Exposed Infants (n = 13)	Maturity-Matched Controls (n = 39)
Neonatal characteristics		
Gestational age, weeks	30 (28–32)	30 (28–32)
Birth weight, g	1286 (958–1453)	1244 (964–1445)
Discharge weight, g	2714 (2380–2980)	2626 (2416–2843)
Weight gain during hospitalization, g/day	20 (18–22)	20 (17.5–22.4)
Days to reach 100 mL/kg/day enteral feeding	8 (6–10)	8 (6–12)
Male sex, n (%)	5 (38.5%)	15 (38.5%)
Small for gestational age, n (%)	6 (46.2%)	16 (41.0%)
5-min Apgar score	8 (6–8)	8 (7–9)
Morbidity		
Respiratory distress syndrome, n (%)	8 (61.5%)	26 (66.6%)
Chronic lung disease, n (%)	10 (76.9%)	23 (59.0%)
Necrotizing enterocolitis, n (%)	0 (0%)	0 (0%)
Sepsis, n (%)	3 (23.1%)	4 (10.3%)
Retinopathy of prematurity > Stage 2, n (%)	3 (23.1%)	8 (20.5%)
Late-onset circulatory collapse, n (%)	2 (15.3%)	2 (5.1%)
Clinical course		
Length of hospital stay, days	83 (37–86)	67 (40–100)
Intubation, n (%)	9 (69.2%)	24 (61.5%)
Mechanical ventilation, days	5 (0–11)	6 (0–18)
Oxygen therapy, days	54 (30–76)	38 (15–64)
Phototherapy, days	4 (3–6)	5 (3–7)

Data are presented as median (interquartile range [IQR]) for continuous variables and number (percentage) for categorical variables. Baseline characteristics and clinical outcomes are presented descriptively. SLE, systemic lupus erythematosus.

## Data Availability

The data supporting the findings of this study are available from the corresponding author upon reasonable request, subject to approval by the Institutional Review Board of the University of Occupational and Environmental Health, Japan, and applicable institutional regulations. The data are not publicly available because they contain potentially identifiable maternal and neonatal clinical information and are subject to privacy and ethical restrictions.

## Supplementary Materials

The following supporting information can be downloaded at: https://www.mdpi.com/article/10.3390/children13091237/s1, Table S1: Hepatic parameters in preterm infants born to mothers with and without SLE; Table S2: Neonatal hepatic parameters according to maternal SLE flare status and maturity-matched controls; Table S3: Maternal SLE-related characteristics and pregnancy management corresponding to SLE-exposed infants; Table S4. Neonatal Hematologic and Hepatic Profiles by Maternal Anti-Ro Autoantibody Status.
