# Supplementary material for: Early Leukocyte Profiles in Preterm Infants Born to Mothers with Systemic Lupus Erythematosus: An Exploratory Matched Cohort Study
Source: Children (Basel). 2026 Sep 12;13(9):1237. doi: 10.3390/children13091237 (PMC13605091; doi:10.3390/children13091237)
Supplement: Supplementary file 1 [file children-13-01237-s001.zip › children-4504251-supplementary.pdf]

Supplementary Table S1. Hepatic and Immunoglobulin Parameters in Preterm Infants Born to Mothers With and Without SLE

**Parameter**	**SLE-exposed** (n=13)	**Controls** (n=39)	**Difference (95% CI)** SLE − control	**P value**
**Parameters at Birth (Day 0)**				
Total bilirubin (mg/dL)	2.6 (2.3–2.9)	2.2 (1.7–2.4)	0.47 (0.14 to 0.80)	0.006
AST (U/L)	30 (25–38)	33 (25–49.5)	−9.59 (−22.69 to 3.51)	0.15
ALT (U/L)	5 (4–5)	3 (2–4)	0.48 (−0.68 to 1.65)	0.41
**Parameters at Postnatal Day 5**				
Total bilirubin (mg/dL)	6.4 (4.5–10.8)	5.5 (4.0–8.0)	1.17 (−0.62 to 2.97)	0.19
AST (U/L)	19 (16–22)	20 (17–26.5)	−2.50 (−8.69 to 3.70)	0.42
ALT (U/L)	6 (4–8)	5 (4–7)	0.46 (−1.26 to 2.17)	0.59
**Peak During Hospitalization**				
Total bilirubin (mg/dL)	7.8 (4.9–10.9)	7.7 (5.7–8.8)	0.23 (−1.62 to 2.08)	0.81
AST (U/L)	43 (37–54)	49.5 (44–82.8)	−32.71 (−89.67 to 24.26)	0.25
ALT (U/L)	24 (21–29)	24.5 (19–34)	−13.34 (−48.42 to 21.75)	0.45

Data are presented as median (interquartile range). Differences are mean group differences with 95% confidence intervals estimated using matched-set fixed-effects linear models; negative values indicate lower values in the SLE-exposed group. Row-specific analytic sample sizes are shown as n=SLE/control.
All analyses were exploratory; no adjustment was made for multiple comparisons, and P values should be interpreted as hypothesis-generating.
SLE, systemic lupus erythematosus; AST, aspartate aminotransferase; ALT, alanine aminotransferase; CI, confidence interval.

Supplementary Table S2. Neonatal Hepatic and Immunoglobulin Parameters According to Maternal SLE Relapse Status and Maturity-Matched Controls

Parameter	Relapse (n=6)	Matched controls (n=18)	Difference (95% CI) relapse − control	P value	Non-relapse (n=7)	Matched controls (n=21)	Difference (95% CI) non-relapse − control	P value
**Parameters at Birth (Day 0)**								
Total bilirubin (mg/dL)	2.5 (2.4–2.6)	2.0 (1.6–2.4)	0.43 (−0.11 to 0.97)	0.11	2.6 (2.2–3.4)	2.2 (1.8–2.4)	0.51 (0.06 to 0.96)	0.03
AST (U/L)	31.5 (22–40.2)	36.5 (26–52.2)	−12.94 (−36.11 to 10.22)	0.25	30 (28.5–32.5)	31 (24.2–44)	−6.66 (−22.51 to 9.19)	0.39
ALT (U/L)	3.5 (3–4)	2.5 (2–3)	0.61 (−0.73 to 1.96)	0.35	5 (5–5)	4 (3–5)	0.37 (−1.60 to 2.34)	0.70
**Parameters at Postnatal Day 5**								
Total bilirubin (mg/dL)	4.1 (3.6–4.9)	4.3 (3.8–5.0)	−0.55 (−3.06 to 1.95)	0.64	10.8 (7.5–12.0)	7.8 (5.9–8.7)	2.86 (0.32 to 5.41)	0.03
AST (U/L)	17 (13.2–24.5)	17 (12.2–21.5)	−1.12 (−13.29 to 11.05)	0.85	19 (18.5–21.5)	24 (18–27)	−3.79 (−8.95 to 1.36)	0.13
ALT (U/L)	4 (4–7)	4 (2.2–5.5)	1.42 (−1.76 to 4.60)	0.35	6 (5.5–7)	7 (5–8)	−0.45 (−2.11 to 1.20)	0.56
**Peak During Hospitalization**								
Total bilirubin (mg/dL)	5.0 (2.3–7.6)	6.3 (4.7–7.8)	−1.22 (−3.52 to 1.09)	0.28	10.8 (7.1–12.0)	8.4 (7.2–9.0)	1.48 (−1.42 to 4.39)	0.30
AST (U/L)	44.5 (42–53)	58.5 (43.2–90.5)	−48.56 (−167.69 to 70.58)	0.40	43 (37–52.5)	47.5 (44–61.8)	−18.90 (−59.33 to 21.53)	0.34
ALT (U/L)	27 (24.8–40.5)	24.5 (19.5–35.5)	−17.28 (−87.95 to 53.39)	0.61	23 (18.5–26)	25 (19–31.5)	−9.90 (−40.72 to 20.91)	0.51

Data are presented as median (interquartile range). Each relapse subgroup was compared only with its independently maturity-matched controls. Differences are mean group differences with 95% confidence intervals estimated using matched-set fixed-effects linear models; negative values indicate lower values in the SLE-exposed subgroup. Row-specific analytic sample sizes are shown as n=SLE/control.

No direct inferential comparison was made between relapse and non-relapse infants. All subgroup analyses were exploratory; no adjustment was made for multiple comparisons.

SLE, systemic lupus erythematosus; AST, aspartate aminotransferase; ALT, alanine aminotransferase; CI, confidence interval.

Supplementary Table S3. Maternal SLE-related characteristics and pregnancy management corresponding to SLE-exposed infants (n=13)

Characteristic	Value
**Baseline maternal characteristics**	
Maternal age, years	30 (27–35)
Prepregnancy SLEDAI	0 (0–4)
Prepregnancy comorbidities and organ involvement	
Lupus nephritis	12 (92.3)
Antiphospholipid syndrome[1]	4 (30.8)
Hypertension	1 (7.7)
Diabetes mellitus	0 (0.0)
Thyroid disease	2 (15.4)
**SLE activity during pregnancy**	
Peak SLEDAI during pregnancy[2]	0 (0–9)
SLE relapse during pregnancy[3]	6 (46.2)
**Pregnancy-related complications**	
Preeclampsia	5 (38.5)
Hypertensive disorders of pregnancy[4]	9 (69.2)
Thrombotic microangiopathy	1 (7.7)
Thrombocytopenia	5 (38.5)
Severe thrombocytopenia (<100,000/µL)[5]	1 (7.7)
**Immunologic profile**	
Anti-Ro antibody	11 (84.6)
Anti-La antibody	6 (46.2)
Anti-RNP antibody	7 (53.8)
Anti-dsDNA antibody	4 (30.8)
Lupus anticoagulant	4 (30.8)
Anticardiolipin antibody	2 (15.4)
Decreased complement levels	2 (15.4)
**Treatment during pregnancy**	
Systemic corticosteroids	8 (61.5)
Hydroxychloroquine	10 (76.9)
Low-dose aspirin	5 (38.5)
Biologic agents[6]	4 (30.8)
**Obstetric management**	
Mode of delivery	
Vaginal delivery	1 (7.7)
Cesarean delivery	12 (92.3)
**Transfusion during pregnancy**	
Red blood cells	5 (38.5)
Platelets	6 (46.2)
Fresh frozen plasma	2 (15.4)

Data are presented as median (interquartile range) or n (%), as appropriate. The unit of presentation is the SLE-exposed infant to align with the neonatal analytic cohort; maternal characteristics from the same pregnancy are repeated for each included infant in a multiple pregnancy.SLE, systemic lupus erythematosus; SLEDAI, Systemic Lupus Erythematosus Disease Activity Index; RNP, ribonucleoprotein; dsDNA, double-stranded DNA.

[1] Antiphospholipid syndrome was recorded according to the diagnosis documented in the medical record.

[2] Peak SLEDAI was the highest value recorded during pregnancy.

[3] SLE relapse during pregnancy was defined as an increase of ≥4 points in SLEDAI from the prepregnancy baseline and/or clinician-documented worsening requiring escalation of immunosuppressive therapy.

[4] Preeclampsia is included within hypertensive disorders of pregnancy and is also shown separately.

[5] Severe thrombocytopenia is a subset of thrombocytopenia.

[6] Biologic agents refer to biologic therapies recorded during pregnancy.

Supplementary Table S4. Neonatal Hematologic and Hepatic Profiles by Maternal Anti-Ro Autoantibody Status

Parameter	Anti-Ro positive, n=11 Median (IQR)	Anti-Ro negative, n=2 Individual values
**Neonatal and maternal characteristics**		
Gestational age, weeks	29 (28–32)	33, 30
Birth weight, g	1286 (852–1400)	1602, 1065
Maternal SLE flare, n (%)	6 (54.5)	0 (0)
**Parameters at Birth (Day 0)**		
White blood cell count (×10³/μL)	6.2 (2.4–8.4)	17.7, 6.4
Absolute neutrophil count (/μL)	1400 (698–5841)	10266, 2688
Platelet count (×10³/μL)	231 (207–275)	342, 229
Hemoglobin (g/dL)	14.9 (13.6–17.5)	16.9, 17
**Parameters at Postnatal Day 5**		
White blood cell count (×10³/μL)	6.8 (4.9–9.7)	10.4, 8.7
Absolute neutrophil count (/μL)	3065 (1560–4766)	3744, 2784
Platelet count (×10³/μL)	244 (167–278)	387, 316
Hemoglobin (g/dL)	13 (10.9–17.4)	15.2, 15.2
**Nadir During Hospitalization**		
White blood cell count (×10³/μL)	5.2 (2.4–8.4)	10.4, 5.4
Postnatal day at nadir	5 (0–37)	5, 71
Absolute neutrophil count (/μL)	924 (634–1442)	3744, 594
Postnatal day at nadir	0 (0–8)	5, 11
Platelet count (×10³/μL)	169 (132–275)	342, 229
Postnatal day at nadir	5 (3–20)	0, 0
Hemoglobin (g/dL)	9.1 (8.4–10.8)	11.1, 9.8
Postnatal day at nadir	29 (14–42)	28, 84
**Parameters at Birth (Day 0)**		
Total bilirubin (mg/dL)	2.6 (2.3–2.9)	2.6, 2.3
AST (U/L)	30 (25–39)	33, 32
ALT (U/L)	5 (4–5)	6, 4
**Parameters at Postnatal Day 5**		
Total bilirubin (mg/dL)	5.1(4.1–9.0)	10.9, 8.7
AST (U/L)	19 (15–24)	22, 18
ALT (U/L)	6 (4–7)	8, 5
**Peak During Hospitalization**		
Total bilirubin (mg/dL)	6.4 (4.3–11)	10.9, 8.7
AST (U/L)	47 (42–55)	37, 37
ALT (U/L)	27 (20–37)	23, 23

Data are presented as median (interquartile range [IQR]) for the anti-Ro-positive group and as individual values for the two infants in the anti-Ro-negative group. Individual values for the anti-Ro-negative infants are presented in consistent infant order. Values at birth were obtained within 24 h after delivery; day-5 values were obtained on postnatal day 5 (±1 day); nadirs and peaks were the lowest and highest values, respectively, recorded during NICU hospitalization. No between-group hypothesis testing was performed because only two infants were included in the anti-Ro-negative group; these comparisons are descriptive and exploratory. Antigen-specific results for anti-TROVE2/Ro60 and anti-TRIM21/Ro52 were not separately available. NICU, neonatal intensive care unit; AST, aspartate aminotransferase; ALT, alanine aminotransferase.
